# Exploring Natural Immune Responses to *Shigella* Exposure Using Multiplex Bead Assays on Dried Blood Spots in High-Burden Countries: Protocol From a Multisite Diarrhea Surveillance Study

**DOI:** 10.1093/ofid/ofad650

**Published:** 2024-03-25

**Authors:** Prisca Benedicto-Matambo, Lindsay N Avolio, Henry Badji, Rabab Batool, Farhana Khanam, Stephen Munga, Milagritos D Tapia, Pablo Peñataro Yori, Alex O Awuor, Bubacarr E Ceesay, Jennifer Cornick, Nigel A Cunliffe, Paul F Garcia Bardales, Christopher D Heaney, Aneeta Hotwani, Mahzabeen Ireen, Md Taufiqul Islam, Ousman Jallow, Robert W Kaminski, Wagner V Shapiama Lopez, Victor Maiden, Usman Nurudeen Ikumapayi, Ruth Nyirenda, John Benjamin Ochieng, Richard Omore, Maribel Paredes Olortegui, Patricia B Pavlinac, Nora Pisanic, Firdausi Qadri, Sonia Qureshi, Nazia Rahman, Elizabeth T Rogawski McQuade, Francesca Schiaffino, Ousman Secka, Catherine Sonye, Shazia Sultana, Drissa Timite, Awa Traore, Mohammad Tahir Yousafzai, Md Taufiqur Rahman Bhuiyan, M Jahangir Hossain, Khuzwayo C Jere, Margaret N Kosek, Karen L Kotloff, Farah Naz Qamar, Samba O Sow, James A Platts-Mills

**Affiliations:** School of Biomedical Sciences and Health Professions, Department of Medical Laboratory Sciences, Kamuzu University of Health Sciences, Blantyre, Malawi; Malawi Liverpool Wellcome Programme, Blantyre, Malawi; Institute of Infection, Veterinary and Ecological Sciences, University of Liverpool, Department of Clinical Infection, Microbiology and Immunology, Liverpool, UK; Department of Environmental Health & Engineering, Johns Hopkins Bloomberg School of Public Health, Baltimore, Maryland, USA; Medical Research Council Unit The Gambia at the London School of Hygiene and Tropical Medicine, Fajara, The Gambia; Department of Pediatrics and Child Health, The Aga Khan University, Karachi, Pakistan; Infectious Diseases Division, International Centre for Diarrhoeal Disease Research, Bangladesh, Dhaka, Bangladesh; Kenya Medical Research Institute, Center for Global Health Research (KEMRI-CGHR), Kisumu, Kenya; Center for Vaccine Development and Global Health, University of Maryland School of Medicine, Baltimore, Maryland, USA; Department of Pediatrics, University of Maryland School of Medicine, Baltimore, Maryland, USA; Department of Medicine, University of Maryland School of Medicine, Baltimore, Maryland, USA; Division of Infectious Diseases and International Health, University of Virginia, Charlottesville, Virginia, USA; Kenya Medical Research Institute, Center for Global Health Research (KEMRI-CGHR), Kisumu, Kenya; Medical Research Council Unit The Gambia at the London School of Hygiene and Tropical Medicine, Fajara, The Gambia; Malawi Liverpool Wellcome Programme, Blantyre, Malawi; Institute of Infection, Veterinary and Ecological Sciences, University of Liverpool, Department of Clinical Infection, Microbiology and Immunology, Liverpool, UK; Institute of Infection, Veterinary and Ecological Sciences, University of Liverpool, Department of Clinical Infection, Microbiology and Immunology, Liverpool, UK; Asociación Benéfica PRISMA, Iquitos, Peru; Department of Environmental Health & Engineering, Johns Hopkins Bloomberg School of Public Health, Baltimore, Maryland, USA; Department of Pediatrics and Child Health, The Aga Khan University, Karachi, Pakistan; Infectious Diseases Division, International Centre for Diarrhoeal Disease Research, Bangladesh, Dhaka, Bangladesh; Infectious Diseases Division, International Centre for Diarrhoeal Disease Research, Bangladesh, Dhaka, Bangladesh; Medical Research Council Unit The Gambia at the London School of Hygiene and Tropical Medicine, Fajara, The Gambia; Latham Biopharm Group, Massachusetts, USA; Asociación Benéfica PRISMA, Iquitos, Peru; Malawi Liverpool Wellcome Programme, Blantyre, Malawi; Medical Research Council Unit The Gambia at the London School of Hygiene and Tropical Medicine, Fajara, The Gambia; Malawi Liverpool Wellcome Programme, Blantyre, Malawi; Kenya Medical Research Institute, Center for Global Health Research (KEMRI-CGHR), Kisumu, Kenya; Kenya Medical Research Institute, Center for Global Health Research (KEMRI-CGHR), Kisumu, Kenya; Asociación Benéfica PRISMA, Iquitos, Peru; Department of Global Health, University of Washington, Seattle, Washington, USA; Department of Environmental Health & Engineering, Johns Hopkins Bloomberg School of Public Health, Baltimore, Maryland, USA; Infectious Diseases Division, International Centre for Diarrhoeal Disease Research, Bangladesh, Dhaka, Bangladesh; Department of Pediatrics and Child Health, The Aga Khan University, Karachi, Pakistan; Infectious Diseases Division, International Centre for Diarrhoeal Disease Research, Bangladesh, Dhaka, Bangladesh; Department of Epidemiology, Emory University, Atlanta, Georgia, USA; Division of Infectious Diseases and International Health, University of Virginia, Charlottesville, Virginia, USA; Faculty of Veterinary Medicine, Universidad Peruana Cayetano Heredia, Lima, Peru; Medical Research Council Unit The Gambia at the London School of Hygiene and Tropical Medicine, Fajara, The Gambia; Kenya Medical Research Institute, Center for Global Health Research (KEMRI-CGHR), Kisumu, Kenya; Department of Pediatrics and Child Health, The Aga Khan University, Karachi, Pakistan; Centre pour le Développement des Vaccins du Mali, Bamako, Mali; Centre pour le Développement des Vaccins du Mali, Bamako, Mali; Department of Pediatrics and Child Health, The Aga Khan University, Karachi, Pakistan; Infectious Diseases Division, International Centre for Diarrhoeal Disease Research, Bangladesh, Dhaka, Bangladesh; Medical Research Council Unit The Gambia at the London School of Hygiene and Tropical Medicine, Fajara, The Gambia; Malawi Liverpool Wellcome Programme, Blantyre, Malawi; Institute of Infection, Veterinary and Ecological Sciences, University of Liverpool, Department of Clinical Infection, Microbiology and Immunology, Liverpool, UK; School of Life Sciences & Health Professions, Department of Medical Laboratory Sciences, Kamuzu University of Health Sciences, Blantyre, Malawi; Division of Infectious Diseases and International Health, University of Virginia, Charlottesville, Virginia, USA; Center for Vaccine Development and Global Health, University of Maryland School of Medicine, Baltimore, Maryland, USA; Department of Pediatrics, University of Maryland School of Medicine, Baltimore, Maryland, USA; Department of Medicine, University of Maryland School of Medicine, Baltimore, Maryland, USA; Department of Pediatrics and Child Health, The Aga Khan University, Karachi, Pakistan; Centre pour le Développement des Vaccins du Mali, Bamako, Mali; Division of Infectious Diseases and International Health, University of Virginia, Charlottesville, Virginia, USA

**Keywords:** diarrhea, dried blood spot, immune response, multiplex bead assay, *Shigella*

## Abstract

**Background:**

Molecular diagnostics on human fecal samples have identified a larger burden of shigellosis than previously appreciated by culture. Evidence of fold changes in immunoglobulin G (IgG) to conserved and type-specific *Shigella* antigens could be used to validate the molecular assignment of type-specific *Shigella* as the etiology of acute diarrhea and support polymerase chain reaction (PCR)–based microbiologic end points for vaccine trials.

**Methods:**

We will test dried blood spots collected at enrollment and 4 weeks later using bead-based immunoassays for IgG to invasion plasmid antigen B and type-specific lipopolysaccharide O-antigen for *Shigella flexneri* 1b, 2a, 3a, and 6 and *Shigella sonnei* in *Shigella*-positive cases and age-, site-, and season-matched test-negative controls from all sites in the Enterics for Global Health (EFGH) *Shigella* surveillance study. Fold antibody responses will be compared between culture-positive, culture-negative but PCR-attributable, and PCR-positive but not attributable cases and test-negative controls. Age- and site-specific seroprevalence distributions will be identified, and the association between baseline antibodies and *Shigella* attribution will be estimated.

**Conclusions:**

The integration of these assays into the EFGH study will help support PCR-based attribution of acute diarrhea to type-specific *Shigella*, describe the baseline seroprevalence of conserved and type-specific *Shigella* antibodies, and support correlates of protection for immunity to *Shigella* diarrhea. These insights can help support the development and evaluation of *Shigella* vaccine candidates.

## Teaser

Multiplex bead-based immunoassays for IgG to conserved and type-specific *Shigella* antigens will be performed on acute and convalescent dried blood spots to characterize conserved and type-specific immune responses and validate molecular identification of type-specific *Shigella* diarrhea.

The recent application of molecular diagnostics to studies of diarrhea etiology in children in low-resource settings has revealed a substantially higher burden of *Shigella* than previously appreciated by culture [1, 2]. The additional episodes detected by polymerase chain reaction (PCR) have been shown to be of similar severity, suggesting that they are clinically relevant [3]. Molecular detection of *Shigella* is being considered as the microbiologic end point for vaccine trials [4]. However, additional confidence in the clinical relevance and microbiologic specificity of these additional molecular detections is critical to both support the burden case and establish PCR as a reliable method for *Shigella* detection for pivotal studies [5]. One possible independent diagnostic gold standard that could be used to support the attribution of *Shigella* as the cause of diarrhea when detected by PCR is serum antibody response. In this manuscript, we will introduce the use of serologic assays for *Shigella*, including specifically the use of multiple bead-based assays on dried blood spots, discuss what is known about the serum antibody response, and describe our planned analyses in the Enterics for Global Health (EFGH)–*Shigella* surveillance study.

##  

### Natural Immunity to *Shigella* Infection and Possible Correlates of Protection for Shigellosis

Humoral responses induced in *Shigella* infection are primarily directed at the lipopolysaccharide (LPS) O-antigen and the invasion plasmid antigens (Ipa) [6]. Studies conducted in *Shigella*-infected Swedish patients reported that anti-LPS and anti-Ipa immunoglobulin G (IgG) responses are good indicators of recent and previous infections, respectively [7]. Invasion plasmid B (IpaB) appears to be a particularly well-conserved and immunogenic antigen, and antibodies to IpaB are a potential correlate of protection [8, 9]. An association between higher levels of IgG1 antibodies with previous exposure to *Shigella* and lower risk of developing symptomatic infection was shown in a study conducted in the Israeli Defense Force using the double antibody sandwich enzyme-linked immunosorbent assay (ELISA) [10]. Anti-LPS antibodies were shown to be a useful diagnostic method in detecting *S*. *flexneri* infection in Vietnamese children aged <3 years [11].

Higher levels of serum IgG antibodies to *Shigella* were measured in subpopulations of high endemic regions, with an increased risk of exposure to *Shigella* in various epidemiological and human challenge studies. Individuals repeatedly infected with *Shigella* acquire immunological correlates of protection against shigellosis that prevent or reduce severity of illness following subsequent infection [12]. The prevalence of anti-*Shigella* LPS antibodies is inversely correlated with age-specific incidence as the pathogen-specific host defenses, absent during early infancy, gradually increase with age [13]. However, this natural immunity attained by preexisting IgG anti-LPS may be serotype-specific [13]. The kinetics of the various immunoglobulins, assessed by ELISA over a 10-week period following the onset of disease, revealed that serum IgG levels tend to peak at 3–4 weeks and decline subsequently at the late convalescent stage, when IgG levels reduced to half compared with early convalescence, but remained higher than the baseline titers [14].

### Serological Testing Using Dried Blood Spots

Use of dried blood spots (DBS) for immunologic surveillance has recently gained attention, particularly in resource-limited settings where logistics and parental preference strongly favor fingerstick sampling to venous sampling [15]. DBS is becoming an indispensable specimen for serological assays as it offers several unique advantages including easiness of collection, storage, shipment, and transportation compared with standard collection methods for venous blood samples, while retaining downstream assay performance and precision [15–18]. This includes quantitative assessment of antibody levels; for example, a recent multicountry study estimating typhoid incidence from community-based serosurveys using models of antibody kinetics used DBS [19]. This performance appears to be independent of the subsequent assays used to measure antibody levels. Excellent correlation was observed between serum and DBS for measurement of anti-*Shigella* antibodies by ELISA, and DBS showed excellent precision and reproducibility using multiplex bead assays [18], but there is a need for additional validation studies [20].

### Advantages and Disadvantages of Multiplex Assays

ELISAs are the standard method for measuring antibody responses but only assess 1 antigen at a time, rendering them costly and labor-intensive, with a large sample needed to measure multiple analytes. Multiplexed immunoassays allow for the detection of multiple antigen-specific antibody responses simultaneously, thus decreasing time, labor, and material expenses [15]. Multiple targets can also be measured from a small sample volume [21], which can allow for less invasive sample collection procedures, including pricks rather than phlebotomy. Further, multiplexing reduces measurement errors and biases because all data collected from each sample are exposed to the same assay conditions. Multiplexing can also reduce human error as there are fewer wells and plates to handle. Finally, multiplex capabilities present the opportunity to consider a variety of types of infectious disease responses simultaneously, applied to measure force of infection and disease dynamics across multiple pathogen types. Together with other methods of exposure assessment, including clinical and environmental surveillance, multiplex immunoassays can help fill the gaps to clarify the scope of disease burden in a population [15, 21, 22].

However, one potential disadvantage of multiplexed immunoassay platforms is cross-reactivity. As antibody responses to multiple antigens are measured simultaneously, it is necessary to select antigens that are highly specific to the pathogen of interest to prevent undesired antibody binding to nontargeted antigens. Another challenge of multiplexing is the need to consider variable dynamic ranges of antibody responses, resulting in differing optimal sample dilutions for different antigens [23]. Further, while the cost per analyte is usually less expensive than in ELISA platforms, the higher upfront cost of the hardware instruments and reagents may be restrictive when establishing a multiplexing method for the first time [24]. Thus, the cost savings are most prominent when multiplexing a large number of targets, testing a large number of samples, or both. Finally, the development of new assays requires an investment of time and technical expertise in individual laboratories.

There are 2 options for multiplex antibody detection assays: bead-based and multiarray electrochemiluminescence. Bead-based immunoassays utilize uniquely labeled microspheres (“beads”) that can be coated with the analyte of interest, allowing the capture and detection of antibodies specific to that analyte (Luminex Corp). In contrast, in multiarray electrochemiluminescence, the analyte of interest is printed in spots on the bottom of a 96- or 384-well plate, with up to 10 spots per well (Meso Scale Diagnostics [MSD]). The Luminex bead-based multiplex assay relies on suspension reaction kinetics of mixing samples with microspheres, allowing for faster, more consistent results than solid phase assays [24]. The Luminex platform can also support multiplexing hundreds of analytes and allows for more flexible selection of assay manufacturers, while the MSD multiarray supports only 10 analytes per well and less accessible assay development. However, the Luminex platform is liable to more variation in plate-to-plate replicability, especially in complex sample matrices like saliva, has a smaller dynamic range, and requires more regular instrument maintenance [24]. Ultimately, assay availability, cost, and instrument availability and prior experience were key factors that led to the selection of a bead-based approach for the EFGH study.

## PROJECT OBJECTIVES

As described elsewhere, *Shigella* spp. will be identified and serotyped in EFGH by both culture and quantitative PCR (qPCR) from whole stools and/or rectal swabs [25, 26]. DBS will be collected via heel or finger prick at enrollment (acute) and 4 weeks later (convalescent). In this exploratory study, we will perform multiplex bead assays on acute and convalescent DBS from children with *Shigella* detected by any method as well as 1:1 age-, site-, and season-matched *Shigella*-negative children to measure IgG to IpaB as well as LPS O-antigen specific to *Shigella sonnei* and *Shigella flexneri* serotypes. Testing of these samples will add several critical pieces to the study to help inform *Shigella* vaccine development. The objectives for this project are as follows:

1. to validate and assess interlaboratory performance of *Shigella* multiplex bead antibody assays;
2. to validate qPCR as a microbiologic end point for phase 3 *Shigella* vaccine trials;
3. to validate *Shigella* serotyping by qPCR directly from stool;
4. to describe the sero-epidemiology of age- and site-specific preexisting immunity against *Shigella*;
5. to evaluate homotypic and heterotypic protection against shigellosis.

## LABORATORY METHODS

### Collection, Processing, Transportation, and Storage of Dried Blood Spot

Dried bloodspot collection is summarized elsewhere [27]. At least 3 fully saturated blood spots will be collected on Whatman 903 Protein Saver Cards and placed at 4°C for storage. Previous studies have shown that antibodies are stable at this temperature for at least 90 days and likely much longer [28, 29].

### Development and Selection of Multiplex Bead Assays for *Shigella* Antibodies

A *Shigella*-specific multiplex bead assay will be developed in collaboration with Luminex Corporation and the Gates Medical Research Institute using unique fluorescently labeled carboxylated magnetic MagPlex microspheres (Luminex Corp). This multiplex assay will include a recombinant IpaB antigen as the broadest marker of prior *Shigella* infection. The multiplex assay will also be designed to allow for serological typing of *Shigella* species by including type-specific LPS antigens from *S*. *flexneri* 1b, 2a, 3a, and 6 and *S*. *sonnei*. These *Shigella* types circulate frequently in the study regions and are considered important strains for candidate vaccine development [30]. This assay is an extension of a previously developed assay that included IpaB, *S. sonnei*, and *S. flexneri* 2a and that was validated against ELISA assays, with a similar approach taken to add additional *S. flexneri* LPS antigens [31]. The LPS antigens will be modified to facilitate coupling to beads that are designed for coupling with peptide-based antigens. The modification method and antigen coupling concentration will be optimized individually for each of the LPS antigens included in the multiplex. The multiplex assay will also include various internal assay control beads, including a bead coupled with antihuman IgG to ensure the quality of the assay as well as a bead coated with bovine serum albumin (BSA) to measure nonspecific binding within individual samples. DBS sample dilution will be optimized to fit the linear range of antibody signal produced by the multiplex assay, and the assay will be measured on Luminex xMAP instruments. Because of the cost and the training and staffing that would be required to test a relatively small number of samples at each site, assays will be performed in Malawi (for samples from the 4 African sites), Bangladesh (for samples from the Bangladesh site), and the University of Virginia (for samples from the Pakistan and Peru sites).

### Assessment of Intra- and Interlaboratory Performance

To ensure consistency and reproducibility of results between laboratories, each laboratory will receive the same coupled bead batches, control material, and detection antibody lots. Matched cases and controls as well as repeated time points by child will be run on the same plate to avoid any plate effects. After training, and periodically as needed, assay performance of each laboratory will be assessed using a *Shigella*-specific sera reference panel with predetermined ranges of acceptable variation. Further, control wells included on each plate will serve as a plate-specific quality control check, a measure of intra-assay lab performance to monitor for any systemic drift in signal over time and allow for additional assessment of performance between laboratories. Additionally, a subset of samples will be tested in duplicate on the same plate to assess intra-assay precision. Finally, a subset of samples from each laboratory site will be sent to a reference laboratory (Johns Hopkins University) to determine the interlaboratory performance across all sites. The intra- and interlaboratory variability will be determined by calculating the standard deviation and percent coefficient of variation (%CV) for each *Shigella* antigen.

## ANALYTICAL METHODS

### Sample Selection and Testing

All proposed analyses will be performed within the same sample selection and study design: a nested case–control study. Cases will consist of all children with *Shigella* detected from rectal swabs by culture or qPCR at any quantity, while test-negative controls will be selected from children who presented with an acute diarrheal illness but did not have *Shigella* detected by any method and matched by site, age, and season. Based on data from the Antibiotics for Children with severe Diarrhoea (ABCD) study, Global Enteric Multicentre Study (GEMS) study, and Malnutrition and Enteric Disease Study (MAL-ED), we conservatively estimate that 25% of children will have *Shigella* detected by culture and/or PCR at least once during the study period, and thus (with 1:1 matching) about 700 children will be included in this substudy from each site (half of the anticipated ∼1400 enrollment target over 2 years). As DBS will be collected upon enrollment and 4 weeks later, we anticipate testing 1400 DBS (700 children × 2 samples) per site. DBS will be identified and tested in 2 batches, approximately corresponding to the first and second year of surveillance.

To define the interlab reproducibility of these assays, we will also select a subset of ∼10% of samples and ship these to the reference laboratory to perform repeat assays, with sample selection designed to represent a range of mean fluorescence intensity (MFI) values for each of the assay targets. Specifically, we will perform stratified random selection of samples for each target and predefine the MFI range (based on the total MFI distribution for each target). We will use the first year of surveillance to identify these samples, to front-load these additional shipments and testing. Approximately 10% same- and between-plate duplicates will also be included to allow for an assessment of intralaboratory performance.

### Analysis

To determine intra- and interlaboratory performance, we will calculate standard metrics of repeatability and coefficients of variation for repeat samples tested within each laboratory as well as for the subset of samples that undergo testing at the reference laboratory. All beads for each antigen will be coupled in a single batch to improve reproducibility and reliability of cross-site measures. Bead performance will also be fully characterized including reproducibility, linearity, repeatability, and precision.

To better understand the clinical relevance and specificity of molecular detection of *Shigella*, we will compare antibody responses after *Shigella* diarrhea. Specifically, we will categorize all diarrhea with *Shigella* detected into culture-positive (regardless of qPCR result), culture-negative/qPCR-attributable (based on the qPCR quantification cycle cutoff developed for EFGH) [26], and culture-negative/qPCR-detected but not attributable (all DNA quantities below the quantitative cutoff). These will be compared with the *Shigella*-negative controls. We will fit a model to estimate the association between *Shigella* attribution category (with the matched controls as the referent) and fold-change in MFI, controlling for age, site, and baseline MFI. Our hypothesis is that culture-positive and culture-negative/qPCR-attributable shigellosis will be associated with a similar immune response (confirming that these are all episodes of shigellosis), but that other *Shigella* detections will not. To interrogate the relevance of qPCR detection below the attribution cutoff for *Shigella* infections, a group for which some residual benefit of azithromycin was seen in the ABCD trial [32], we will also model immune response as it relates to *Shigella* cycle threshold values, accounting for age and site, to independently ascertain a qPCR cutoff that can be compared with the EFGH prespecified cutoff. Next, we will evaluate, for both isolate-based serotyping and qPCR-based serotyping, the association between type-specific infection and type-specific antibodies. Defining type-specific infection by immune response alone, we can evaluate the relative specificity of serotype assignment by culture and qPCR. While heterotypic responses are expected, the homotypic response should be strongest. We will follow a previous approach used for norovirus genotype assignment by serologic studies [33]. These analyses will help establish the clinical relevance of qPCR ascertainment of *Shigella* infection as well as qPCR-based speciation and serotyping.

To describe the site- and age-specific prevalence of antibodies to conserved and type-specific *Shigella* infections in the EFGH study, we will use only the acute DBS samples (collected at enrollment), which should reflect preexisting *Shigella* antibodies rather than a response to the current infection. To make the acute case–control samples selected for testing more representative of all children enrolled in EFGH, we will apply inverse probability of selection weights based on a model where the outcome is the probability of selection for testing and predictors include detection of *Shigella*, site, age, year, and calendar month. This will re-inflate the *Shigella*-negative samples to make the overall estimates more representative. We will then describe age-specific antibody MFI distributions for each target. To calculate seroprevalence, we will first have to establish MFI cutoffs to define seropositivity. The most common approach, identified in a recent review of this challenge, is to define a population cutoff using presumed unexposed individuals [34]. This is expected to be a post hoc analysis, identifying a subpopulation that is expected to have little or no *Shigella* exposure, for example, children 6–9 months of age without *Shigella* detected by any method. We will then use the MFI distribution in these children to establish a cutoff that will be applied to all other age strata. Alternatively, it may be preferable for the cutoffs to be generated for each site, but this will require some visual inspection of the data and will require a sufficient amount of data points at the site level. Once a cutoff or cutoffs have been established, we will describe the weighted prevalence of antibodies to conserved and type-specific antigens by age and site. This will help predict the proportion of children with preexisting immunity to *Shigella* in clinical trials, which can then be a planned subgroup analysis in phase 3 trials.

To understand the specificity of the antibody response to type-specific *Shigella* infections, we will subset to *Shigella*-positive children who (a) have a specific *Shigella* type identified by culture and/or qPCR and (b) have a ≥4-fold MFI response to ≥1 conserved *Shigella* antigen, with post hoc sensitivity analyses for alternative changes in MFI. We will then describe the distribution in type-specific fold MFI changes between enrollment (acute) and 4-week (convalescent) DBS and evaluate for the breadth of MFI changes for each type. To define the degree of homotypic and heterotypic protection from antibodies to *Shigella*, we will then use the test-negative design to evaluate the association between the presence of conserved and type-specific *Shigella* antibodies at baseline and *Shigella* diarrhea as well as *S*. *flexneri* serotypes or *S*. *sonnei* detected in the diarrheal sample [35]. Specifically, we will estimate the association between the baseline quantity and presence of conserved and type-specific *Shigella* antibodies and *Shigella* attribution, adjusting for potential predictors of *Shigella* infection including age, season, and sociodemographic markers. Among *Shigella* cases, we will fit a second model to estimate the association between the quantity and presence of conserved and type-specific *Shigella* antibodies and *Shigella* species and serotype. This will help define whether these IgG responses are a correlate of protection for natural immunity.

#### Challenges and Strengths for Inclusion of Serologic Assays in EFGH

Several possible risks and challenges are important to consider. First, it is possible that the assays will not perform as expected. If analyses raise questions about assay performance, and because the IpaB assay is particularly critical for the proposed analyses, we will consider performing a single plex ELISA assay for this target on a subset of samples to further evaluate and validate the multiplex bead assay results. Estimates of baseline seroprevalence using samples from children presenting with diarrhea may partially represent an early response to the acute infection. In a previous facility-based diarrhea surveillance study, ∼95% of enrolled children had ≤5 days of symptoms at enrollment, by which time IgG antibody responses would be expected to be minimal [36]. It is also possible that children will have preexisting elevated levels of *Shigella* antibodies, making it more difficult to detect differences between the levels measured in the acute and convalescent samples. Since the EFGH study will enroll children from 6 to 35 months of age, including the peak age of *Shigella* diarrhea, the detected cases will likely present with an initial episode of *Shigella* [37]. Moreover, as symptoms are less likely with a subsequent infection, these episodes are less likely to meet eligibility criteria [38]. Finally, the exclusion of children under 6 months of age should minimize the presence of maternal antibodies [39]. The timing of convalescent samples is also important when interpreting these data; we plan to collect these 4 weeks after the time of enrollment, which is the expected peak of serum IgG responses [36]. IgG levels have been noted to remain elevated up to 10 weeks and support the occurrence of a recent *Shigella* infection in adults [13]. This timing has been used in vaccine trials to support the presence of an immune response, and the expected increase in IgG to acute infection is likely to be multiple-fold [40]. While it is possible that some children will have a new *Shigella* infection during the 4-week window, this is expected to be relatively rare and is unavoidable, as a sufficient window to observe an IgG response is critical. Because the analyses will be performed on large numbers of children with and without shigellosis, we do not anticipate that this will significantly alter the results or interpretation of the planned analyses. Another challenge in data interpretation will be defining seropositivity/response. In the absence of serial samples to define the antibody kinetics, the ability to accurately define seropositivity will be difficult and will likely rely on post hoc analyses and sensitivity analyses to establish robust evidence.

Although there may be challenges to interpreting the immune response data, our study has many strengths that favor the generalizability and relevance of this work. The EFGH study is being conducted in 7 countries on 3 continents in urban and rural settings, resulting in a rich data set that will include a variety of observations that could highlight differences and similarities between the regions. For instance, the epidemiology and age of acquisition of *Shigella* among the study sites may differ [41]. Shared standardized procedures will ensure that interpretation is not confounded by differences in specimen collection or timing. From a logistic perspective, DBS collection is preferred over venipuncture for compliance with this study procedure and will ensure a suitable quantity of sample. Use of a multiplex bead assay will ensure our ability to measure these antibodies by minimizing the volume of sample required. Our detection of *Shigella* diarrhea is also optimized using culture and qPCR, which is known to increase the amount of *Shigella*-attributable disease [2].

## CONCLUSIONS

In summary, inclusion of serologic assays on acute and convalescent DBS collected from children enrolled in EFGH opens the opportunity to add a significant additional dimension to our understanding of *Shigella* burden and immune response and can help support the development and evaluation of *Shigella* vaccine candidates.
